# Neutrophil/Lymphocyte Ratio Is Associated with Non-Calcified Plaque Burden in Patients with Coronary Artery Disease

**DOI:** 10.1371/journal.pone.0108183

**Published:** 2014-09-30

**Authors:** Lennart Nilsson, Wouter G. Wieringa, Gabija Pundziute, Marcus Gjerde, Jan Engvall, Eva Swahn, Lena Jonasson

**Affiliations:** 1 Department of Medical and Health Sciences, Linköping University, Linköping, Sweden; 2 Department of Cardiology, Linköping University, Linköping, Sweden; 3 Department of Clinical Physiology, Linköping University, Linköping, Sweden; 4 University of Groningen, University Medical Center Groningen, Department of Cardiology, Groningen, The Netherlands; University of Bologna, Italy

## Abstract

**Background:**

Elevations in soluble markers of inflammation and changes in leukocyte subset distribution are frequently reported in patients with coronary artery disease (CAD). Lately, the neutrophil/lymphocyte ratio has emerged as a potential marker of both CAD severity and cardiovascular prognosis.

**Objectives:**

The aim of the study was to investigate whether neutrophil/lymphocyte ratio and other immune-inflammatory markers were related to plaque burden, as assessed by coronary computed tomography angiography (CCTA), in patients with CAD.

**Methods:**

Twenty patients with non-ST-elevation acute coronary syndrome (NSTE-ACS) and 30 patients with stable angina (SA) underwent CCTA at two occasions, immediately prior to coronary angiography and after three months. Atherosclerotic plaques were classified as calcified, mixed and non-calcified. Blood samples were drawn at both occasions. Leukocyte subsets were analyzed by white blood cell differential counts and flow cytometry. Levels of C-reactive protein (CRP) and interleukin(IL)-6 were measured in plasma. Blood analyses were also performed in 37 healthy controls.

**Results:**

Plaque variables did not change over 3 months, total plaque burden being similar in NSTE-ACS and SA. However, non-calcified/total plaque ratio was higher in NSTE-ACS, 0.25(0.09–0.44) vs 0.11(0.00–0.25), p<0.05. At admission, levels of monocytes, neutrophils, neutrophil/lymphocyte ratios, CD4+ T cells, CRP and IL-6 were significantly elevated, while levels of NK cells were reduced, in both patient groups as compared to controls. After 3 months, levels of monocytes, neutrophils, neutrophil/lymphocyte ratios and CD4+ T cells remained elevated in patients. Neutrophil/lymphocyte ratios and neutrophil counts correlated significantly with numbers of non-calcified plaques and also with non-calcified/total plaque ratio (r = 0.403, p = 0.010 and r = 0.382, p = 0.024, respectively), but not with total plaque burden.

**Conclusions:**

Among immune-inflammatory markers in NSTE-ACS and SA patients, neutrophil counts and neutrophil/lymphocyte ratios were significantly correlated with non-calcified plaques. Data suggest that these easily measured biomarkers reflect the burden of vulnerable plaques in CAD.

## Introduction

Coronary artery disease (CAD) is the leading cause of death in the western world. Although multifactorial in its origin, inflammatory and immunological events are considered to play central roles in initiation and progression of atherosclerotic plaques [1]. Indeed, elevations in soluble markers of inflammation as well as changes in leukocyte subset distribution are frequently reported in patients with CAD [2]–[5]. However, studies on relationships between markers of inflammation and severity of CAD have yielded disparate results [6]–[9].

In recent years, several studies have demonstrated the important role of neutrophils in all stages of atherosclerosis and plaque destabilization leading to acute coronary syndromes (ACS) [10]. Accordingly, neutrophil infiltration has been detected in very early stages of atherosclerosis as well as in shoulder regions of plaques prone to rupture [11], [12]. Circulating neutrophil counts and neutrophil/lymphocyte ratios are emerging markers of the presence and severity of CAD [13]–[15]. Furthermore, they are independent predictors of mortality and cardiovascular events in high-risk groups and a broad range of CAD patients [14], [16]–[19].

CAD severity is not only a question about the extent of obstructive stenosis, but the risk of plaque rupture and ACS largely depends on plaque composition [20]. Coronary computed tomography angiography (CCTA) is a non-invasive method allowing accurate assessment of CAD [21]. In contrast to invasive coronary angiography (ICA), CCTA provides information about the vessel wall and composition of plaques in addition to degree of stenosis. CCTA may therefore provide valuable information about the burden of CAD with prognostic implications as well as assessing the morphological aspects of the disease process [22]–[24]. The identification of circulating immune-inflammatory markers that are associated with the atherosclerotic disease process in coronary arteries may provide additive information. The aim of the study was to investigate if neutrophil counts, neutrophil/lymphocyte ratio or other immune-inflammatory markers were related to plaque burden, as assessed by CCTA in patients with stable angina (SA) and ACS.

## Methods

### Study design and population

This study is a single-center, prospective, pilot study. The study population consisted of 30 patients with SA, 20 patients with non-ST-elevation acute coronary syndrome (NSTE-ACS), and 37 healthy control subjects. In order to assess coronary atherosclerosis the patients underwent CCTA prior to ICA and revascularization (percutaneous coronary intervention (PCI) or coronary artery bypass grafting). A follow-up CCTA was performed at three months following revascularization in order to reevaluate the plaque burden and plaque composition. Blood samples were collected at baseline (prior to CCTA and ICA), and at three months follow-up. Inclusion criteria were as follows: 1) patients with planned ICA due to SA, defined as clinically probable angina pectoris and positive exercise test or myocardial perfusion imaging; 2) NSTE-ACS, defined as unstable angina or non-ST-elevation myocardial infarction, according to universally accepted definitions [25], [26]. The exclusion criteria were: 1) inability to perform CCTA (contraindications to betablockers or nitroglycerine, allergy to contrast medium, pregnancy, permanent atrial fibrillation); 2) renal dysfunction (creatinine >150 µmol/L) or risk factors for contrast induced acute kidney injury (treatment with metformin, high dose diuretics); 3) known factors influencing immunological and inflammatory markers (active immunologic or inflammatory disease, infection with fever or use of antibiotics during the last 30 days, immunosuppressive treatment); and 4) major trauma, surgery or PCI in the last 30 days. The control subjects, randomly invited from the Swedish Population Register and representative for hospital recruitment area, were clinically healthy and received no medication.

### Ethical Considerations

The study protocol was approved by the Regional Ethical Review Board in Linköping and written informed consent was obtained from all study participants. The study was conducted in accordance with the ethical guidelines set forth in the Declaration of Helsinki.

### Coronary computed tomography angiography: image acquisition

Patients underwent CCTA within twenty-four hours after inclusion using a 16-slice multi-slice CT scanner or a 64-slice dual-source CT scanner (Sensation or Somatom Definition, Siemens Healthcare, Forchheim, Germany). During CTA acquisition non-ionic contrast medium was administered (Iomeron 400, Bracco, Altana, Pharma, Konstanz, Germany). Beta-blocker treatment (orally or intravenously) and nitroglycerine was administered to achieve optimal image quality. In order to reduce radiation exposure, electrocardiogram-gated current modulation was used in all patients. The following scan parameters were used: 1. For the 16-slice multi-slice scanner: 16×0.75 mm collimation, gantry rotation time of 375 ms, temporal resolution of 188 ms, tube voltage 100 or 120 kV, and maximal tube current of 650 mAs; 2. For the 64-slice dual-source CT scanner: 64×2×0.6 mm collimation, gantry rotation time of 330 ms, temporal resolution of 83 ms, tube voltage 100 or 120 kV, and maximal tube current of 560 mAs. Upon completion of the scan, images were reconstructed, if possible in several phases of the R-R interval, to obtain motion-free images of the coronary arteries.

### Coronary computed tomography angiography: image analysis

Evaluation of CCTA images was performed on a remote workstation with dedicated software (QAngio CT, Medis Medical Imaging Systems, Leiden, the Netherlands) [27], side by side in consensus by two experienced observers blinded to baseline patient characteristics and ICA results. A predefined window and level setting (window 900 HU, level 250 HU) was used for analysis of lumen and plaque [28]. Coronary segments were differentiated into seventeen segments, according to a modified American Heart Association classification [29]. Segments of insufficient quality for evaluation were scored as non-evaluable and excluded from analysis. The CT scan was considered unevaluable if 2 vessels had 3 or more segments that were non-evaluable. Presence of plaques was visually assessed. Coronary atherosclerosis was defined as tissue structures >1 mm2 within or adjacent to the coronary artery lumen but distinctive from surrounding pericardial or epicardial tissue. Per segment, one coronary plaque was selected. The degree of luminal narrowing of the coronary artery was quantified visually, based on comparison of the luminal diameter of the plaque containing segment to the luminal diameter of the most normal-appearing site immediately proximal to the plaque. Plaques with ≥50% luminal narrowing were classified as obstructive. In addition, plaque composition was assessed. Three types of plaques were classified: 1) Non-calcified plaque (plaques with lower density compared to contrast-enhanced lumen), 2) calcified plaque (plaques with high density structures compared to contrast-enhanced lumen), or 3) mixed plaque (non-calcified and calcified constituents in single plaque) [30]. The number of any plaques (total plaque burden), as well as plaques with different features was calculated per patient.

### Invasive coronary angiography: image acquisition and analysis

Since the assessment of degree of stenosis with 16-slice CCTA is only moderately accurate, ICA was used to assess the obstructive plaque burden. ICA of left and right coronary arteries was performed in multiple views by using the transfemoral approach. Coronary segments were scored in the same manner as on CCTA images, and a diameter stenosis of ≥50% was classified as obstructive. Digital angiograms were analyzed off-line with dedicated software (Coronary Artery Analysis System 9, Pie Medical Imaging, Maastricht, The Netherlands). All segments >1.5 mm in diameter with a <100% diameter stenosis were measured on the angiograms. The contrast-filled non-tapered catheter tip was used for calibration. The proximal and distal reference vessel diameters and minimal lumen diameter of the suspected lesion were recorded. The percentage of diameter stenosis was calculated.

### Blood sampling, biochemical analysis and flow cytometry

Venous blood samples were collected in vacutainer tubes (using sodium heparin as anticoagulant). Baseline blood samples of NSTE-ACS patients were received within 24 hours of hospital admission. For all CAD patients, the samples at baseline and at three months were collected prior to CCTA and ICA. Samples were centrifuged within 30 minutes to separate plasma, which then was stored immediately at −70°C until analyzed. White blood cell differential counts were determined in whole blood by Cell-Dyn Sapphire™ (Abbot Diagnostics). Leukocyte subset distributions were analyzed in whole blood by flow cytometry as previously described [31], [32]. Briefly, monoclonal antibodies against CD3, CD4, CD8, CD19, CD16 and CD56 were purchased from BD Biosciences, San José, CA, US. The antibodies were marked with one of 3 fluorochromes: fluorescein isothiocyanate, phycoerythrin and peridinin chlorophyll protein. The cells were identified by combinations as follows: CD3/CD4/CD8 (T helper cells and cytotoxic T cells), CD19 (B cells) and CD3/CD16/CD56 (NK cells). Whole blood and antibodies were incubated for 15 minutes at room temperature, thereafter erythrocytes were lysed with FACS™ Lysing Solution (BD Biosciences) for 15 minutes at room temperature. Samples were analyzed on a FACSCanto II (BD Biosciences) equipped with 3 lasers, a blue 488 nm, a red 633 nm and a violet 405 nm. Analysis of samples was stopped when 10 000 cells were collected in the lymphocyte gate. Data were analyzed and subpopulations gated with FACSDiva™ 6.1.2 software (BD Biosciences). C-reactive protein (CRP) was measured in serum using a highly sensitive latex-enhanced turbidimetric immunoassay (Roche Diagnostics GmbH, Vienna, Austria) with a lower limit of detection of 0.03 mg/L. IL-6 levels in plasma were measured using an ELISA (R&D Systems Europe, Abingdon, United Kingdom) with a lower limit of detection of 0.48 pg/mL.

### Statistical analysis

Categorical variables are presented as numbers (percentages) and were compared between groups using chi-square or Fisher's exact tests. When normally distributed, continuous variables are expressed as mean ±SD and were compared using one-way ANOVA and Students t-test for independent samples. When non-Gaussian distributed, continuous variables are presented as medians with 25^th^ and 75^th^ percentiles and were compared using the nonparametric Kruskal-Wallis test and Mann-Whitney-U test. Wilcoxon signed-rank test was used for paired comparisons. Bivariate correlations were performed to assess the associations between continuous variables using Spearman's correlation coefficient. A p-value of <0.05 was considered statistically significant. Statistical analyses were performed using SPSS version 20 (Chicago, IL, USA) and STATA version 11.0 (College Station, TX, USA).

## Results

### Study population

During the study period from March 2006 till January 2009 a total of 64 patients were initially included in the study. Fourteen patients were excluded from analysis: 11 patients did not complete any CCTA examination and in 3 patients the CCTA was of non-diagnostic quality. Baseline clinical characteristics of all patients are listed in Table 1. Revascularization was performed in 22 of 30 SA patients (8 PCI and 14 CABG) and in 13 of 20 NSTE-ACS patients (9 PCI and 4 CABG) within the first two months after study inclusion. All CAD patients were treated with statin therapy after admission.

**Table 1:** Baseline clinical and biochemical characteristics of patients and controls.

	SA	NSTE-ACS	Controls	P-value	
	N = 30	N = 20	N = 37		
Age (years)	64±9	67±10	64±8	NS	
Female	4 (13)	5 (25)	9 (24)	NS	
Waist circumference (cm)	102±11	99±13	95±9	0.003^ψ^	
Current or previous smoker	22 (73)	15 (75)	1 (3)	<0.001^ψγ^	
Hypertension	21 (70)	7 (35)	0	<0.001^ψγ^, 0.015^*Φ*^	
Diabetes mellitus	4 (13)	1 (5)	0	0.022^ψ^	
History of MI	6 (20)	2 (10)	0	0.004^ψ^	
Statin treatment	24 (80)	6 (30)	0	<0.001^ψγ*Φ*^	
Plasma cholesterol (mmol/L)	5.1±1.0	5.4±1.3	5.5±1.1	NS	
Plasma LDL-cholesterol (mmol/L)	2.9±0.8	3.2±1.2	3.4±0.9	0.013^ψ^	
Plasma HDL-cholesterol (mmol/L)	1.2±0.2	1.3±0.4	1.4±0.4	0.010^ψ^	
Triglycerides (mmol/L)	1.4 (1.1,2.1)	1.4 (1.2,1.7)	1.1 (0.9,1.6)	0.018^ψ^, 0.038^γ^	

The data are presented as mean ±SD, median (25^th^, 75^th^ percentile), or numbers (%). SA =  stable angina; ACS =  acute coronary syndrome; MI =  myocardial infarction; LDL =  low density lipoprotein, HDL =  high density lipoprotein. NS =  non-significant (p≥0.05). For comparisons between groups, p-values indicating significant differences are denoted by ψ (controls vs SA), γ (controls vs NSTE-ACS) and *Φ* (SA vs NSTE-ACS), respectively.

### Coronary computed tomography angiography

**Figure 1:**
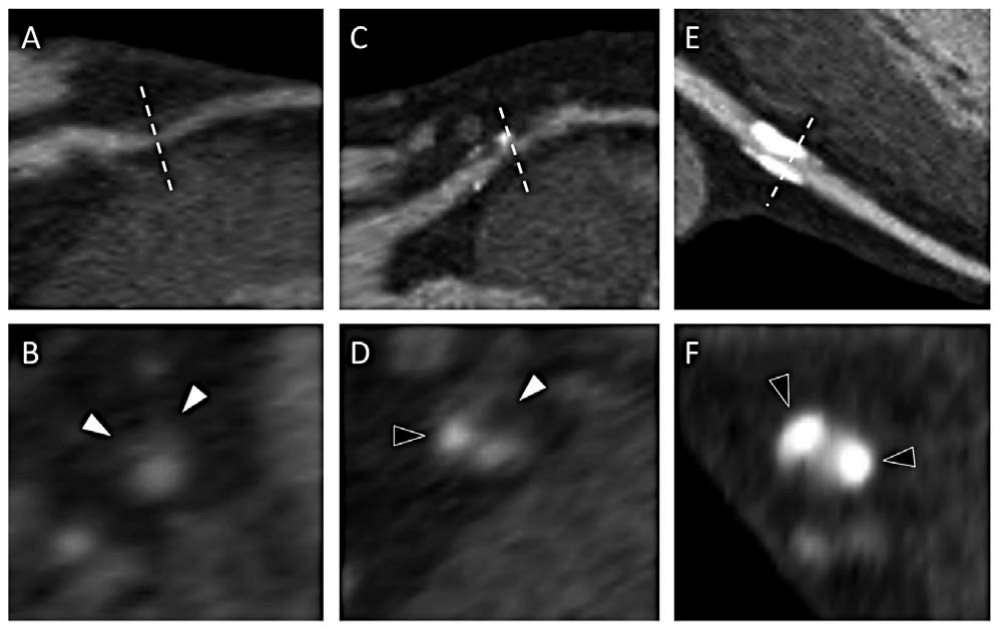
Different plaque compositions as seen by CCTA. The different types of coronary plaque are shown in longitudinal views, with cross-sectional views at the level of the dotted line. A non-calcified plaque is shown on the left (A and B), with white arrowheads pointing at the non-calcified plaque component. A mixed plaque is shown in the middle (C and D), with a white arrowhead indicating the non-calcified plaque component and a black arrowhead indicating the calcified component. A large calcified plaque is shown on the right side (E and F), with black arrowhead indicating the calcifications.

Data of the baseline CCTAs are summarized in Table 2. A 16-slice CCTA was used in 10 and a 64-slice CCTA in 40 of the 50 patients. Typical CCTA images of coronary plaques are shown in Figure 1. The total plaque burden (i.e. the total number of any plaque) did not differ between SA and NSTE-ACS patients neither did plaque characteristics differ significantly between the patient groups. However, there was a trend towards more calcified plaques in SA patients, whereas NSTE-ACS patients tended to have more non-calcified plaques. As a measure of vulnerable plaques, the ratio between non-calcified plaques and total plaques was calculated. This ratio was significantly higher in ACS patients as compared to SA patients.

**Table 2:** Baseline coronary computed tomography angiography and invasive coronary angiography.

	SA; N = 30	NSTE-ACS; N = 20	P-value
**CCTA characteristics at admission**			
Number of segments	15 (15,16)	16 (15,16)	NS
Total plaque burden	9 (6,11)	8 (6,10)	NS
Non-calcified plaque	1 (0,2)	2 (1,4)	NS
Mixed plaque	3 (1,5)	4 (1,6)	NS
Calcified plaque	3 (2,5)	2 (0,3)	NS
Non-calcified & mixed plaque	4 (3,7)	6 (2,8)	NS
Ratio non-calcified plaque/plaque burden	0.13 (0.00,0.25)	0.25 (0.10,0.44)	0.049
**ICA characteristics at admission**			
Number of segments	15 (15,16)	16 (15,16)	NS
Segments with significant stenosis (>50%)	3 (2,6)	2 (1,4)	NS
Segments without significant stenosis	12 (9,14)	14 (11,15)	NS

The data are presented as median (25^th^, 75^th^ percentile). CCTA =  coronary computed tomography angiography; ICA =  invasive coronary angiography; SA =  stable angina; ACS =  acute coronary syndrome. NS =  non-significant (p≥0.05).

Follow-up CCTA at 3 months was available in 41 patients (26 SA and 15 NSTE-ACS patients), 9 performed by a 16-slice CCTA and 32 by a 64-slice CCTA. There were no differences in total plaque burden or plaque characteristics between baseline and 3 months. Total plaque burden and non-calcified plaque/total plaque ratio were 9 (5, 11) and 0.16 (0.00, 0.26), respectively, in SA patients, and 8 (3, 10) and 0.25 (0.09, 0.44), respectively, in NSTE-ACS patients, at 3 months.

### Invasive coronary angiography

The findings on ICA are presented in Table 2. There was no difference in the number of coronary artery segments with obstructive lesions in SA patients as compared to NSTE-ACS patients. Strong positive correlations were found between the number of obstructive stenosis on ICA and total plaque burden as well as number of non-calcified plaques on CCTA (r = 0.694, p<0.001, and r = 0.398, p = 0.004, respectively). On the other hand, the non-calcified plaque/total plaque ratio was not associated with the number of obstructive lesions on ICA (r = 0.163, NS).

### Immune-inflammatory markers

At admission, levels of leukocytes, neutrophils, monocytes, neutrophil/lymphocyte ratios, CD4+ T cells, and plasma levels of CRP and IL-6 were significantly elevated, while levels of NK cells were reduced, in SA and NSTE-ACS patients as compared to controls (Table 3). Plasma CRP in SA and NSTE-ACS patients declined over time reaching similar levels as control subjects at 3 months (0.9 (0.3, 2.8), 0.7 (0.3, 1.9) and 0.7 (0.4, 1.2) ng/mL, respectively, NS). Similarly, plasma IL-6 in SA and NSTE-ACS patients did not differ significantly from controls at follow-up (2.4 (1.1, 2.9), 1.7 (1.2, 3.1) and 1.4 (1.0, 2.2) ng/mL, respectively, NS).

**Table 3:** Baseline leukocyte subsets and plasma cytokines of patients and controls.

	SA	NSTE-ACS	Controls	P-value
	N = 30	N = 20	N = 37	
Leukocytes (x10^9^/L)	7.1±2.2	7.7±2.3	5.5±1.4	<0.001^ψγ^
Neutrophils (x10^9^/L)	4.1±1.5	4.5±1.7	3.0±1.0	<0.001^ψγ^
Monocytes (x10^9^/L)	0.6±0.2	0.6±0.3	0.4±0.1	<0.001^ψγ^
Lymphocytes (x10^9^/L)	2.2±1.0	2.4±0.9	2.0±0.6	NS
CD19+ cells, % of lymphocytes	10 (7,12)	10 (8,15)	10 (7,14)	NS
CD4+ cells, % of lymphocytes	49 (42,54)	50 (44,54)	39 (33,48)	0.005^ψ^, 0.001^γ^
CD8+ cells, % of lymphocytes	25 (20,31)	25 (17,31)	23 (19,29)	NS
NK cells, % of lymphocytes	12 (9,18)	12 (7,15)	18 (11,28)	0.023^ψγ^
Neutrophil/lymphocyte ratio	2.1 (1.4,2.4)	2.0 (1.5,2.5)	1.5 (1.2,1.9)	0.012^ψ^, 0.027^γ^
Plasma IL-6, pg/mL	3.1 (2.0,5.7)	4.9 (2.9,8.0)	1.4 (1.0,2.2)	<0.001^ψγ^
Plasma CRP, mg/L	1.2 (0.3,3.2)	3.2 (2.2,5.0)	0.7 (0.4,1.2)	0.023^*Φ*^, <0.001^γ^

The data are presented as mean ±SD or median (25^th^, 75^th^ percentile). SA =  stable angina; NSTE-ACS =  non-ST-elevation acute coronary syndrome; CD19+ cells, B cells; CD4+ cells, T helper cells; CD8+ cell, cytotoxic T cell; NK =  Natural killer; IL =  interleukin; CRP =  C-reactive protein. NS =  non-significant (p≥0.05). For comparisons between groups, p-values indicating significant differences are denoted by ψ (controls vs SA), γ (controls vs NSTE-ACS) and *Φ* (SA vs NSTE-ACS), respectively.

At 3 months, neutrophil counts were still significantly higher in SA and NSTE-ACS patients as compared to control subjects (4.0±1.6, 4.0±1.4 and 3.0±1.0×10^9^/L, respectively, p = 0.006), as were the neutrophil/lymphocyte ratios (2.0±0.8, 2.2±0.7 and 1.5±0.7, respectively, p = 0.030). Also, levels of monocytes and CD4+ T cells remained unchanged at 3 months whereas NK cell levels increased (data not shown). Of note, SA and NSTE-ACS patients did not differ significantly in any immune-inflammatory markers at 3 months.

### Correlations of coronary computed tomography angiography and invasive coronary angiography with immune-inflammatory markers

**Figure 2:**
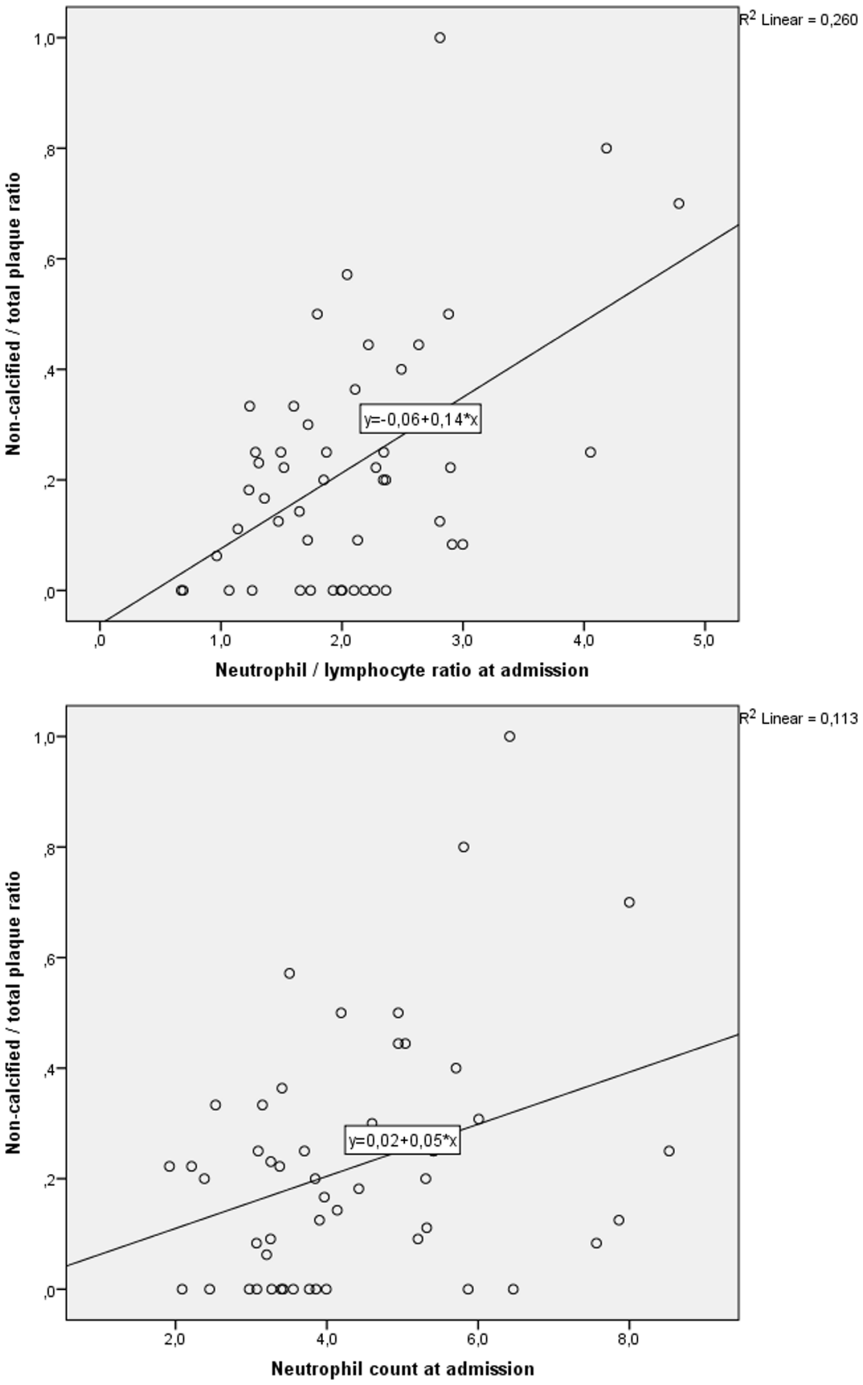
Neutrophils and coronary plaques on CCTA. Scatterplots demonstrating the associations between non-calcified/total plaque ratio on baseline CCTA and neutrophil/lymphocyte ratio and neutrophil counts at admission. Neutrophil counts are given as ×10^9^/L.

Neutrophil/lymphocyte ratios and neutrophil counts correlated with numbers of non-calcified plaques (r = 0.302, p = 0.028 and r = 0.327, p = 0.017) and also with non-calcified plaque/total plaque ratio (r = 0.403, p = 0.010 and r = 0.382, p = 0.024, respectively), but not with total plaque burden on CCTA (Figure 2). Furthermore, neutrophil/lymphocyte ratios and neutrophil counts did not correlate with number of obstructive lesions on ICA. Other leukocyte subsets did not show any correlations with any of the plaque variables, neither did CRP or IL-6 (data not shown).

## Discussion

This study investigated associations between immune-inflammatory markers and plaque burden, as assessed by CCTA in patients with stable and unstable conditions of CAD. The main finding of this study was the consistent and significant correlation of neutrophil counts and neutrophil/lymphocyte ratios with numbers of non-calcified plaques and non-calcified plaque/total plaque ratio, but not with total plaque burden.

Neutrophils and neutrophil/lymphocyte ratios were significantly higher in both SA and NSTE-ACS patients as compared to controls, not only at admission but also at 3 months. The contribution of immune cells such as monocytes/macrophages and T cells to atherosclerosis and plaque progression has been firmly established over the years [33]. However, while being the most abundant white blood cell in the circulation, neutrophils are rarely detected in atherosclerotic plaques and therefore have attracted less attention. Nevertheless, over the past couple of years numerous studies have lent support to an important role of neutrophils in all stages of atherosclerosis [10]. Neutrophil count and neutrophil/lymphocyte ratio are emerging markers of presence and severity of CAD [13]–[15], [34]. They are both independent predictors of mortality and future cardiovascular events in healthy populations, high-risk groups and a broad range of CAD patients [14], [16]–[19], [35], [36].

Neutrophil counts and neutrophil/lymphocyte ratios have been associated with the presence, severity and progression of coronary atherosclerosis as assessed by various modes of coronary imaging [14], [15], [37]. In a large cohort study of 3005 consecutive patients undergoing ICA for various indications, neutrophil count and neutrophil/lymphocyte ratio correlated significantly with the number of diseased vessels [14]. Moreover, a higher neutrophil/lymphocyte ratio was associated with higher coronary calcium scores measured by multidetector CT in 849 clinically healthy individuals participating in a health promotion program [37]. However, neither ICA nor coronary calcium score, provide any information about the plaque composition. Correlations of neutrophil counts and neutrophil/lymphocyte ratios with plaque composition on CCTA have not previously been performed.

By using CCTA it has been possible to show that plaque morphology is an independent predictor of prognosis [22], [24], [38]. Several studies have found that non-calcified plaques are associated with worse outcomes [23], [39]–[40]. Among 3,499 consecutive symptomatic SA patients who underwent CCTA, 1,102 subjects with non-obstructive CAD were prospectively followed for a mean of 78 months. The death rate of these patients was 3.1%, increasing incrementally from calcified plaque (1.4%) to mixed plaque (3.3%) to non-calcified plaque (9.6%) [23]. Our present data indicate that the absolute neutrophil count as well as the neutrophil/lymphocyte ratio, obtained by a white blood cell differential test, reflects the burden of high-risk plaques, rather than the crude number of plaques. Combining these easily available biomarkers and CCTA may be of supplemental value in the identification of patients with vulnerable atherosclerotic plaques. It opens up the potential for early selective treatment and prevention of future myocardial damage.

The increased levels of CD4+ T cells and dynamic changes of NK cells in the patient population of the present study have been described recently [32], [41]. However, we did not find any association between these cell subsets and CCTA plaque characteristics. Neither were there any relationships between the well-established inflammatory markers, CRP and IL-6, and the CCTA findings. Only one previous CCTA study has investigated immune cells in relation to plaque burden and plaque composition. Kashiwagi et al found that an increased level of the proinflammatory CD14+ CD16+ monocyte subset, but not the total number of monocytes or CRP, is related to the presence of vulnerable plaques in patients with stable angina pectoris [42]. Unfortunately, we did not measure monocyte subsets, but the lack of association between total number of monocytes, CRP and the presence of vulnerable plaques on CCTA was also found in our study.

A few studies have been performed in search of associations between other inflammatory markers and CCTA characteristics [43], [44]. Bamberg et al determined the association between several plasma biomarkers and coronary plaque burden assessed by CCTA in 313 patients with acute chest pain who ultimately had no evidence of ACS [43]. Only 25% of study individuals were on statins and those with prior CAD were excluded. Interestingly, they found higher levels of CRP and oxidized LDL cholesterol and lower levels of adiponectin in patients with exclusively non-calcified plaques as compared to those with any calcified plaque or no plaque at all. In our study, 60% of all patients were on statin treatment at the time of inclusion. This may explain the absence of correlations between CRP and plaque characteristics, since statins are known to markedly reduce CRP levels [45]. Another study by Harada et al included 178 non-ACS acute chest pain patients, who underwent CCTA examination [44]. In contrast to the study by Bamberg et al, they found an association between CRP and the presence of calcified rather than non-calcified plaques.

This study has some major limitations. First, the size of the study population was small. On the other hand, we included both SA and ACS patients, who were examined by CCTA and blood sample analysis prior to ICA and revascularization as well as in the stabilized phase at 3 months. Secondly, this study was performed between 2006 and 2009, first using a 16-slice and later a 64-slice CCTA. The 16-slice CCTA is less accurate than the 64-slice CCTA in measuring degree of stenosis and plaque composition. However, we did use ICA as well to assess obstructive and non-obstructive plaque burden and CCTA was performed twice in a majority of patients yielding identical results. Thus, in order not to reduce the size of the study population, all study patients irrespective of CT-scanner used were included in the data analysis.

To conclude, both neutrophil counts and neutrophil/lymphocyte ratio were significantly correlated with non-calcified plaque burden and non-calcified plaque/total plaque ratio. The results highlight the potential utility of these easily measured cellular markers in the risk assessment and monitoring of CAD patients.
